# Predicting mortality in intensive care unit patients with CAUTI using an interpretable machine learning model: a retrospective cohort study from MIMIC-IV database

**DOI:** 10.3389/fmed.2025.1665035

**Published:** 2025-09-09

**Authors:** Longcha Liu, Xueshu Yu, Zhi Chen, Qixia Zhang, Danwen Zhuang

**Affiliations:** Department of Intensive Care Unit, The First Affiliated Hospital of Wenzhou Medical University, Wenzhou, China

**Keywords:** CAUTI, mortality, intensive care unit, prediction, logistic regression, SHAP

## Abstract

**Objective:**

The aim of this study was to develop a reliable model for predicting mortality in patients with catheter-associated urinary tract infection (CAUTI) in intensive care unit (ICU).

**Methods:**

The MIMIC-IV database was used for model development and validation in this study. Data from the first 24 h of ICU admission were collected, and 70% of the data were used to train the model and 30% to validate the model. Four machine learning models, including XGBoost, DecisionTree (DT), Logistic Regression (LR) and Random Forest (RF), were used to construct the prediction model. The SHAP method was used to explain the best performance model.

**Results:**

A total of 545 patients with CAUTI were finally included. The mortality of ICU patients with CAUTI was 7.89% (43/545). The area under the curve (AUC) of the Logistic regression model was 0.871, which showed better prediction performance among the four models. The DecisionTree machine had limited generalization ability, with an AUC of 0.542 and relatively poor prediction accuracy. The SHAP technique revealed 13 most important predictors of CAUTI in order of importance, among which use of vasoactive drugs,shock index,APSIII score, and concomitant malignancy were identified as variables with high predictive significance.

**Conclusion:**

The interpretable prediction model used in this study can help medical staff improve their ability to predict the risk of death in patients with CAUTI in ICU.

## Introduction

Catheter-associated urinary tract infection (CAUTI) is one of the most common health care-associated infections in critical care settings worldwide. Epidemiological studies have shown that (1) the incidence of CAUTI varies significantly across different healthcare systems and economic contexts, ranging from 1.3 to 8.9 per 1000 catheter days. Critical CAUTI is closely related to poor outcomes. In low- and middle-income countries (LMICs), the mortality rate of CAUTI is as high as 31.14%. Studies have highlighted that CAUTI prolongs the average length of hospital stay of patients by 17.84 days and generates an additional cost of approximately US $1,006 per case in the United States (1–3). Studies have suggested (4, 5) that once a patient is diagnosed with CAUTI, the risk of related death is about 10%, which brings heavy clinical and economic burden, and the incidence of infection in ICU increases sharply, which significantly affects the prognosis of patients. As the risk of infection increases, especially in critically ill patients, it is critical to accurately predict the risk of CAUTI related mortality, as this information is essential for clinical decision making and appropriate resource allocation (6, 7).

In recent years, the effectiveness of machine learning (ML) in the field of healthcare prediction has been well demonstrated, such as designing a predictive model for prolonged length of stay (LOS) of extremely preterm infants (vpi) for risk management and decision aid in the early postpartum period (8). And machine learning analytics to diagnose and predict the incidence of pneumonia in patients undergoing elective cardiac surgery (9). Given the inherent ability of machine learning algorithms to capture non-linear relationships, more and more researchers advocate the development of new predictive models to improve patient treatment outcomes.

The purpose of our study is to use the Medical Information Database for Intensive Care (MIMIC-IV) to integrate key clinical variables and develop an interpretable model to predict the risk of death in patients with catheter-associated urinary tract infection (CAUTI) in the intensive care unit (ICU). In addition, SHapley Additive exPlanations (SHAP) method was used to explain the model and explore the prognostic factors of CAUTI. Our study provides reference for clinical medical staff by deeply exploring the risk factors related to death. By identifying poor prognostic outcomes in patients at an early stage of the disease, timely interventions can be taken to improve patient survival, and ultimately improve clinical decision-making and patient outcomes (10, 11).

## Materials and methods

### Data source

This retrospective study utilized the Medical Information Intensive Care (MIMIC-IV) database (v3.1), an iterative version following MIMIC-III. The database complies with HIPAA security regulations and ensures anonymization of the data. MIMIC-IV contains a large amount of clinical data from 70,000 adult intensive care unit (ICU) patients at the Boston Diabetes Research Institute (BIDMC) between 2008 and 2019 (12).

All patient data within the database is anonymized, obviating the need for informed consent. In adherence to the ethical standards articulated in the 1964 Declaration of Helsinki and its subsequent amendments, the study was conducted. Access to the database was secured following the completion of the National Institutes of Health Web-based training course and the Protecting Human Research Participants examination (No. 43258214).

### Participant selection

Patients fulfilling specific criteria were screened through the MIMIC-IV database (version 3.1) for this study. We identified individuals in the database meeting the following criteria:

(1) patients were diagnosed with CAUTI according to the International Classification of Diseases, as indicated by ICD-9 codes, or ICD-10 codes;

(2) only the initial ICU admission date was considered for patients with multiple ICU admissions;

(3) patients were aged 18 years or older.

**FIGURE 1:**
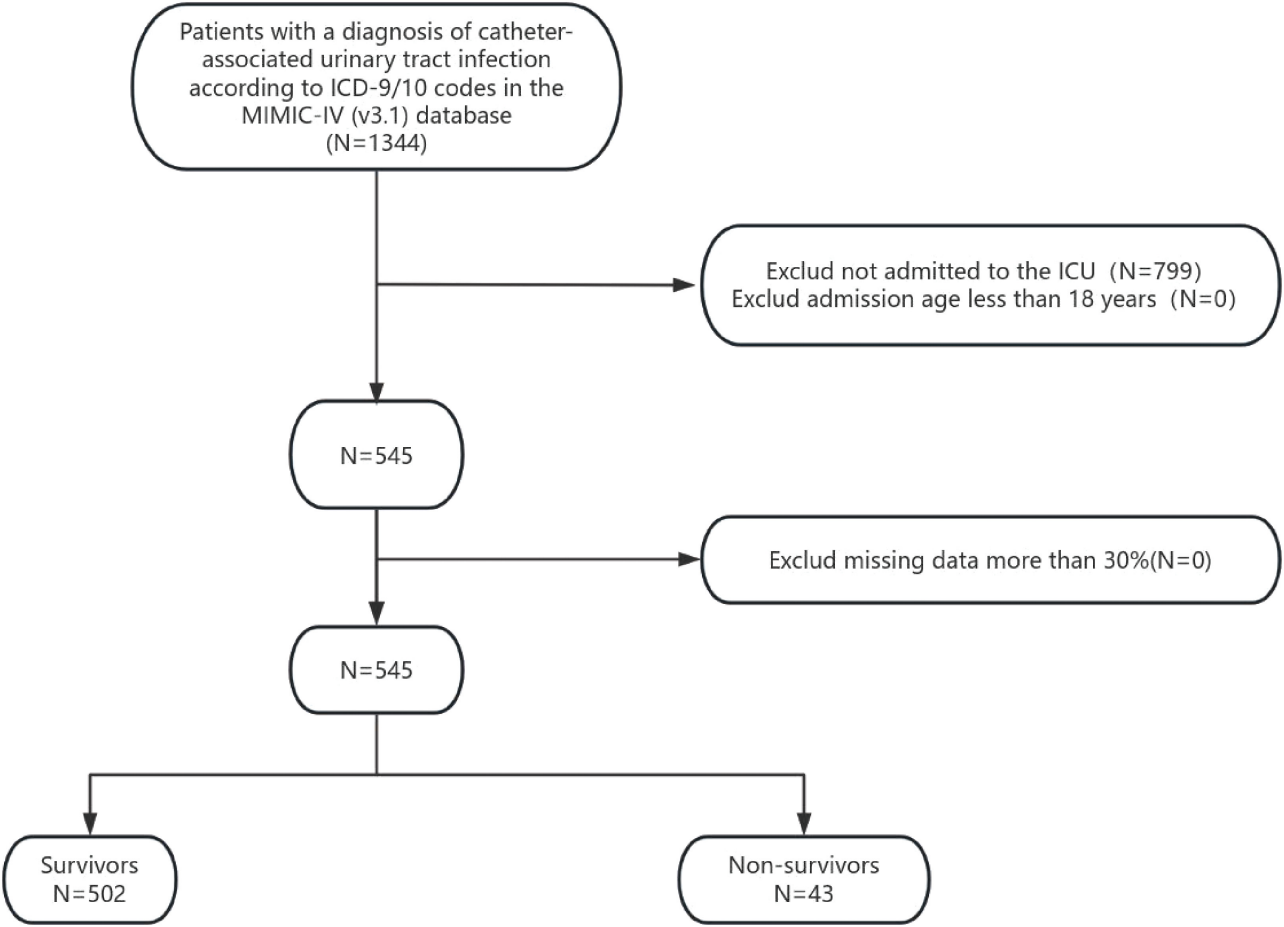
Flowchart for patient selection. ICD, international classification of diseases.

Patients who had more than 30% missing values were excluded (13). Ultimately, 545 patients were enrolled in this study (Figure 1).

### Data extraction, preparation, and definitions

The predicted outcome was the probability of death during a stay in the intensive care unit (ICU). Baseline demographic variables, comorbidities, vital signs, length of hospital stay, severity scores, and laboratory data were extracted from the MIMIC database based on previous studies as well as expert input and implemented using SQL (Structured Query Language) programming. With the exception of length of stay, vital signs were collected within the first 24 h after each ICU admission, whereas other variables were measured at admission. In addition, in order to avoid overfitting, the least absolute shrinkage and selection operator (LASSO) method was used for variable selection and screening, and the LASSO regression was used to select the optimal regularization parameter λ by 10-fold cross-validation (14).

### Management of missing data

Missing data often occur in the MIMIC-IV database. However, if these missing values are ignored during the analysis, the results may be biased. Therefore, we used chained equation multiple imputation (MICE) to deal with missing values, and the number of imputation was set to 5 times to deal with missing data (15). The proportion of missing values in each of the selected variables was less than 30%.

### Machine learning explainable tool

The prediction model is interpreted by SHAPmethod, which is a comprehensive method that can accurately evaluate the contribution and influence of each feature on the final prediction result. SHAP analysis is implemented based on the SHAP 0.44.0 library of Python 3.8 (16). SHAP values indicate the extent to which each predictor variable affects the target variable, either positively or negatively. Furthermore, each data point can be understood by its specific set of SHAP values.

### Statistical analysis

DecisionLinnc1.0 software is used for data analysis, DecisionLinnc1.0 is a platform that integrates multiple programming language environments and realizes data processing, data analysis and machine learning through a visual interface (17). Categorical variables were presented as total amounts and percentages, and the chi-square test or Fisher exact probability method was used to compare the differences between different groups. Continuous variables were expressed as medians and interquartile ranges (IQR), and comparisons between the two groups were performed with the use of the Wilcoxon rank-sum test.

Four machine learning models -XGBoost, DecisionTree (DT), Logistic Regression (LR), and Random Forest (RF) -were used to construct the prediction model. The predictive performance of each model was evaluated by the area under the receiver operating characteristic curve. In addition, we calculated accuracy, precision, and F1 scores. In addition, in order to evaluate the practicality of the model in decision making by quantifying the net benefits under different threshold probabilities, decision curve analysis (DCA) was performed (18).

## Results

### Patient characteristics

In this study, 545 adult patients diagnosed with CAUTI were included out of a total of 1344 patients with CAUTI in the MIMIC-IV database. The depiction of the patient screening process can be observed in Figure 1.

Table 1 presents the baseline characteristics of 545 patients who fulfilled the inclusion criteria, categorized into the ICU survival group and non-survival group. The mortality rate of ICU patients diagnosed with CAUTI was 7.89% (43/545). Among these patients, there were 261 females (47.89%) and 284 males (52.11%), with a median age of 74 (21–99) years, and the age difference was not significant (*P* = 0.803). In terms of length of hospital stay, the median length of hospital stay was 11.94 days for survivors and 15.86 days for non-survivors (*P* = 0.117), and there was no significant difference in length of ICU stay (*P* = 0.065). Severity of illness score showed that SOFA score, APSIII score, APSII score, OASIS score and shock index of non-survivors were significantly higher than those of survivors (*P* < 0.05). The duration of mechanical ventilation of non-survivors was significantly longer (*P* = 0.004), and vital signs such as heart rate, respiratory rate, and blood oxygen saturation showed significant differences. Among the laboratory indicators, lactic acid, PH value, international normalized ratio of prothrombin time and creatinine were significantly poor in non-survivors (*P* < 0.05). In terms of complications, the incidence of acute renal failure was significantly higher in non-survivors, as was the incidence of malignancies. In terms of drug use, the use of sedatives, analgesics and vasoactive drugs in non-survivors was significantly higher than that in survivors (*P* < 0.05). The LASSO regularization method was used to select 13 potential predictors from the training dataset, and these factors were used for model development.

**TABLE 1:** All variables for patients with CAUTI (*N* = 545).

Variable names	Overall	Survivors	Non-survivors	*P*-value
Number	545	502	43	
**Demographics**				
Age(years), median (IQR)	74 (21–99)	75 (21–99)	73 (30–93)	0.803
Gender (male), *n*(%)	284 (52.11)	260 (51.79)	24 (55.81)	0.728
**Length of stay**				
Hosp_day, days	12.19 (0.43–211.14)	11.94 (1.48–161.54)	15.86 (0.43–211.14)	0.117
ICU_day, days	2.52 (0.06–95.84)	2.475 (0.06–95.84)	3.78 (0.36–66.27)	0.065
**Ventilation**				
Ventilation (%)	402 (73.76)	367 (73.11)	35 (81.40)	0.315
Ventilation_hour	18 (0–1692.28)	17.8 (0–1692.28)	26 (0–1541)	0.004
**Comorbidities, *n* (%)**				
Hypertension (%)	203 (37.25)	190 (37.85)	13 (30.23)	0.408
Cancer (%)	104 (19.08)	90 (17.93)	14 (32.56)	0.032
Heart failure (%)	182 (33.39)	162 (32.27)	20 (46.51)	0.083
Acute kidney injury (%)	275 (50.46)	243 (48.41)	32 (74.42)	0.002
Type 1 diabetes mellitus (%)	15 (2.75)	14 (2.79)	1 (2.33)	1.000
Type 2 diabetes mellitus (%)	175 (32.11)	156 (31.08)	19 (44.19)	0.110
**Vital signs, median (IQR)**				
Nibp_mean, mmHg	80 (31–166)	80 (31–166)	79 (35–122)	0.471
Temperature, F	98.2 (86.5–103.4)	98.2 (86.5–103.4)	98.2 (92.9–101.4)	0.186
Heart rate, beats/min	90 (41–178)	89.5 (41–178)	97 (65–137)	0.009
Respiratory rate, times/min	19 (0–50)	19 (0–50)	23 (12–42)	0.001
SpO_2_ (%)	98 (64–100)	98 (64–100)	97 (84–100)	0.042
**Severity scoring**				
SOFA	4 (0–17)	4 (0–17)	7 (1–16)	<0.001
APSIII	46 (13–135)	45 (13–114)	64 (21–135)	<0.001
APSII	38 (6–100)	37 (6–88)	48 (21–100)	<0.001
OASIS	31 (12–56)	31 (12–56)	37 (24–56)	<0.001
GCS	14 (3–15)	14 (3–15)	14 (3–15)	0.159
Charlson comorbidity index	6 (0–17)	6 (0–17)	7 (1–14)	0.034
Shock index	1.071 (0.316–3.812)	1.049 (0.316–3.812)	1.578 (0.611–3.167)	<0.001
**Laboratory variables, median (IQR)**				
Hemoglobin, g/dL	9.8 (2.7–18.8)	9.8 (2.7–18.8)	8.8 (5.9–17.7)	0.024
Red_blood_cells,10^12^L	3.38 (1.36–7.04)	3.39 (1.36–7.04)	3.11 (2.01–5.85)	0.109
White_blood_cells,10^9^/L	10.9 (0.4–66.7)	10.8 (1–66.7)	12.6 (0.4–50.8)	0.114
Rdw (%)	15.3 (11.7–28.8)	15.2 (11.7–28.8)	16.2 (12.5–24.2)	0.001
Platelet_count,10^9^/L	214 (8–847)	213 (25–847)	226 (8–763)	0.906
Hematocrit (%)	30.3 (9.6–58.8)	30.55 (9.6–58.8)	27.3 (19.8–55.3)	0.042
Urea_nitrogen, mg/dL	24 (4–184)	24 (4–184)	42 (9–141)	<0.001
Creatinine, mg/dL	1.1 (0.2–8.7)	1.1 (0.2–8.7)	1.8 (0.4–6.3)	0.008
Alanine_aminotransferase, U/L	21 (3–5970)	20 (3–5970)	25 (4–620)	0.944
Bilirubin_total, μmol/L	0.5 (0.1–17.9)	0.5 (0.1–17.9)	0.5 (0.1–12.9)	0.281
Aspartate_aminotransferase, U/L	30 (6–4494)	30 (6–4494)	32 (11–825)	0.601
Lactate (mmol/L)	1.5 (0.4–12)	1.5 (0.4–11)	1.8 (0.5–12)	0.020
PH	7.38 (6.87–7.62)	7.39 (6.87–7.62)	7.35 (7.06–7.51)	0.025
INR(PT)	1.3 (0.9–7.5)	1.3 (0.9–7.5)	1.4 (1–5.9)	0.005
PT(sec)	14.1 (10.1–82.2)	14 (10.1–82.2)	15.5 (10.9–65.1)	0.002
**Drugs**				
Sedative analgesic (%)	229 (42.02)	202 (40.24)	27 (62.79)	0.007
Vasopressor (%)	229 (42.02)	199 (39.64)	30 (69.77)	<0.001
Glucocorticoids (%)	144 (26.42)	129 (25.70)	15 (34.88)	0.258
Antihypertensive (%)	358 (65.69)	324 (64.54)	34 (79.07)	0.079

Nibp, non-invasive blood pressure; SpO2, O2 saturation; APSIII, acute physiology and chronic health evaluation III; SOFA, sequential organ failure assessment; SAPSII, simplified acute physiology score II; OASIS, oxford acute illness severity score; Rdw, red blood cell distribution width; PT, prothrombin time.

### Model building and evaluation

**FIGURE 2:**
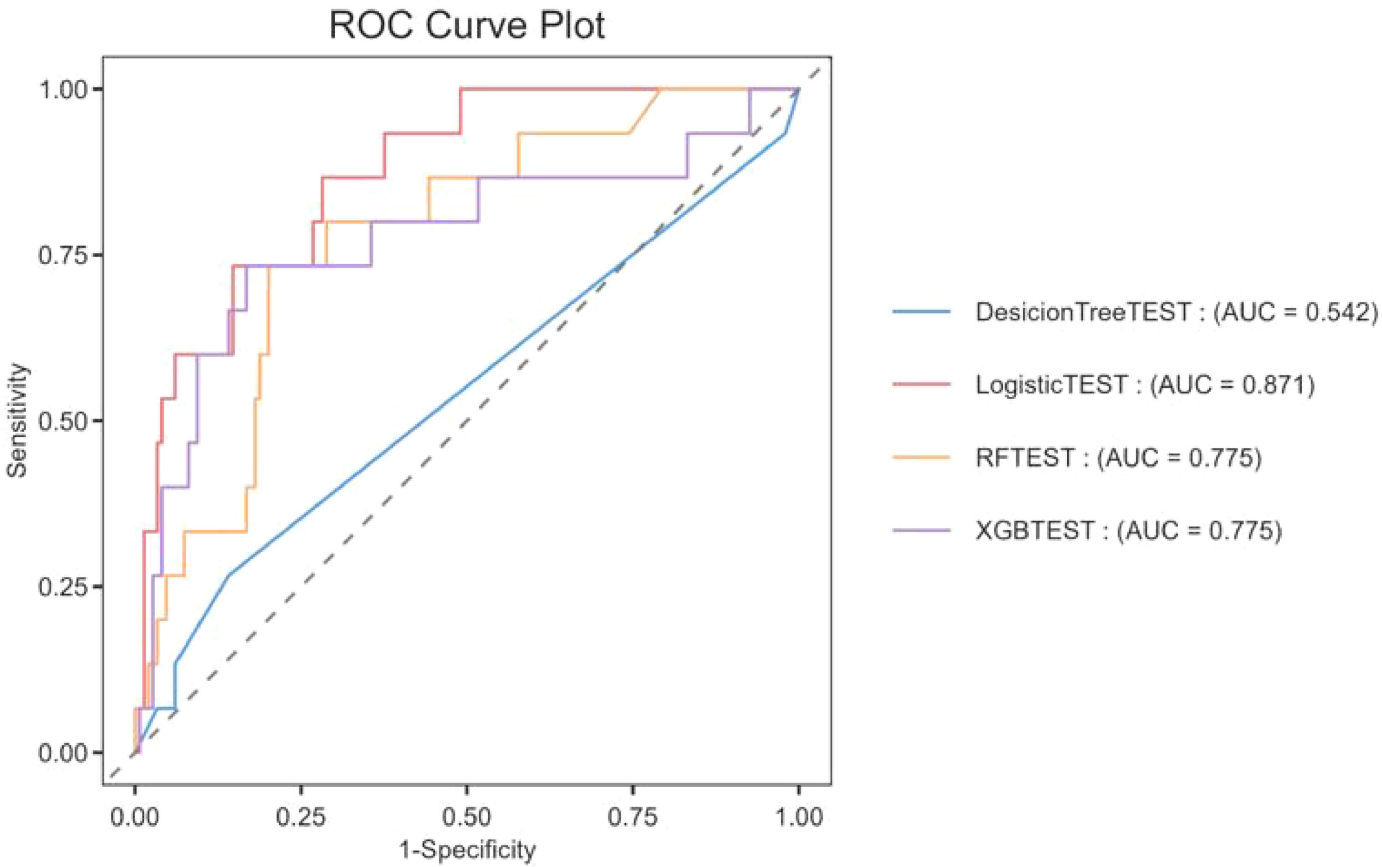
The ROC curve was used to compare the performance of four models in predicting the ICU mortality rate of patients with CAUTI.

The dataset was divided in a random fashion into two parts: 70% of the data was used to train the model, while 30% was used to validate the model. In the training dataset, we built four models: XGBoost, Logistic Regression (LR), Random Forest (RF), and Decision Tree (DT). The AUC values obtained from the test dataset are shown in Figure 2 and Table 2, respectively. Among these models, LR showed superior predictive performance with an AUC of 0.871, while DT had the lowest generalization ability with an AUC of 0.542. The net benefit of the best-performing model was compared with an alternative approach to clinical Decision making using Decision Curve Analysis (DCA) on the test dataset.

**TABLE 2:** Evaluation of predictive performance for each model.

Model name	Accuracy	F1-score	AUROC	Precision
Random forest	0.8963	0.1905	0.7752	0.3333
XGBoost	0.9024	0.3333	0.7749	0.4444
Decision tree	0.8659	0.1538	0.5418	0.1818
Logistic regression	0.9146	0.3	0.8707	0.6

**FIGURE 3:**
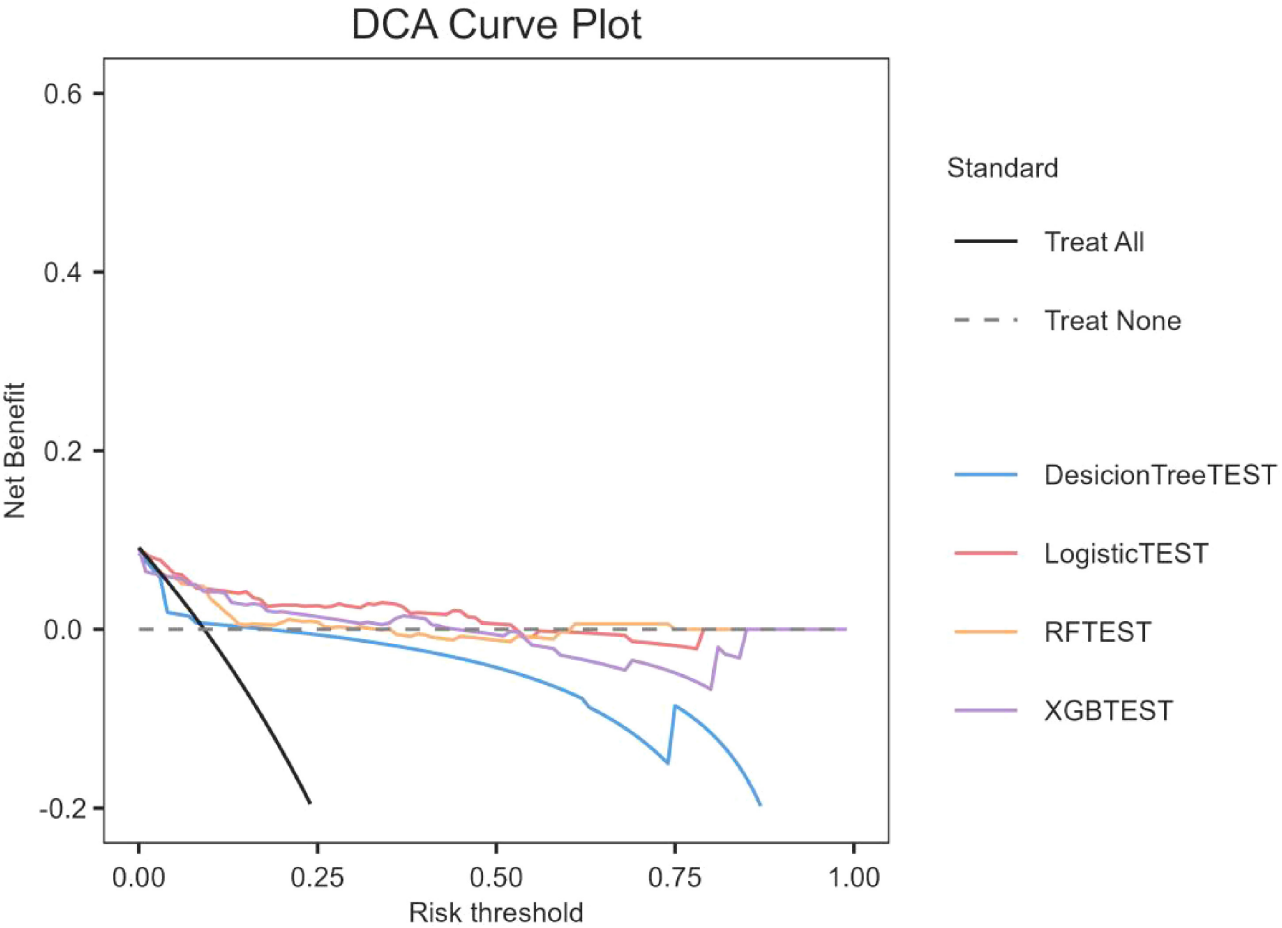
Decision curve analysis of four models plotting the net benefit at different threshold probilities.

We evaluated the overall payoffs at different probability thresholds. The assumptions in Figure 3, represented by the black line, assume that all patients received the intervention. On the other hand, the dashed line represents the case where no patient received any intervention. Given the diverse nature of the study population, developing a treatment strategy based on any of the four machine-learning models would be preferable to treating all or none of the patients by default.

### Explanation model with the SHAP method

The SHAP algorithm was used to determine the importance of each predictor variable in the prediction results of the LR model. The variable importance map presents a list of variables ranked from highest to lowest according to their level of importance.

**FIGURE 4:**
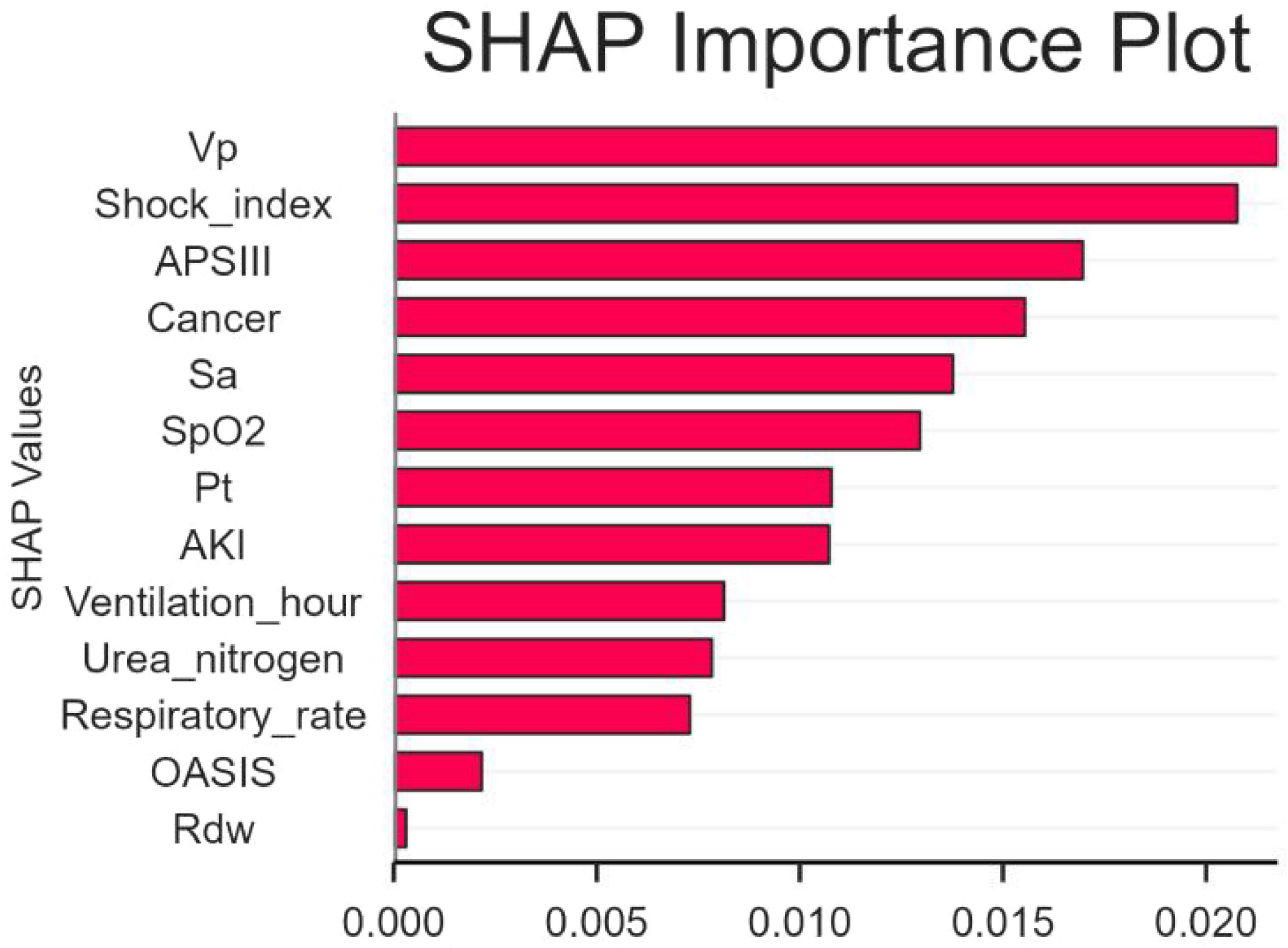
The significance of variable weights. SpO2, O2 saturation; APSIII, acute physiology and chronic health evaluation III; VP, vasopressor; SA, sedative analgesic; PT, prothrombin time; AKI, acute kidney injury; OASIS, oxford acute illness severity score; Rdw, red blood cell distribution width; SHAP, SHapley Additive explanation.

The use of vasoactive agents was considered to have the highest predictive value of all prediction periods, followed by the shock index, coexisting malignancies and APSIII score (Figure 4).

**FIGURE 5:**
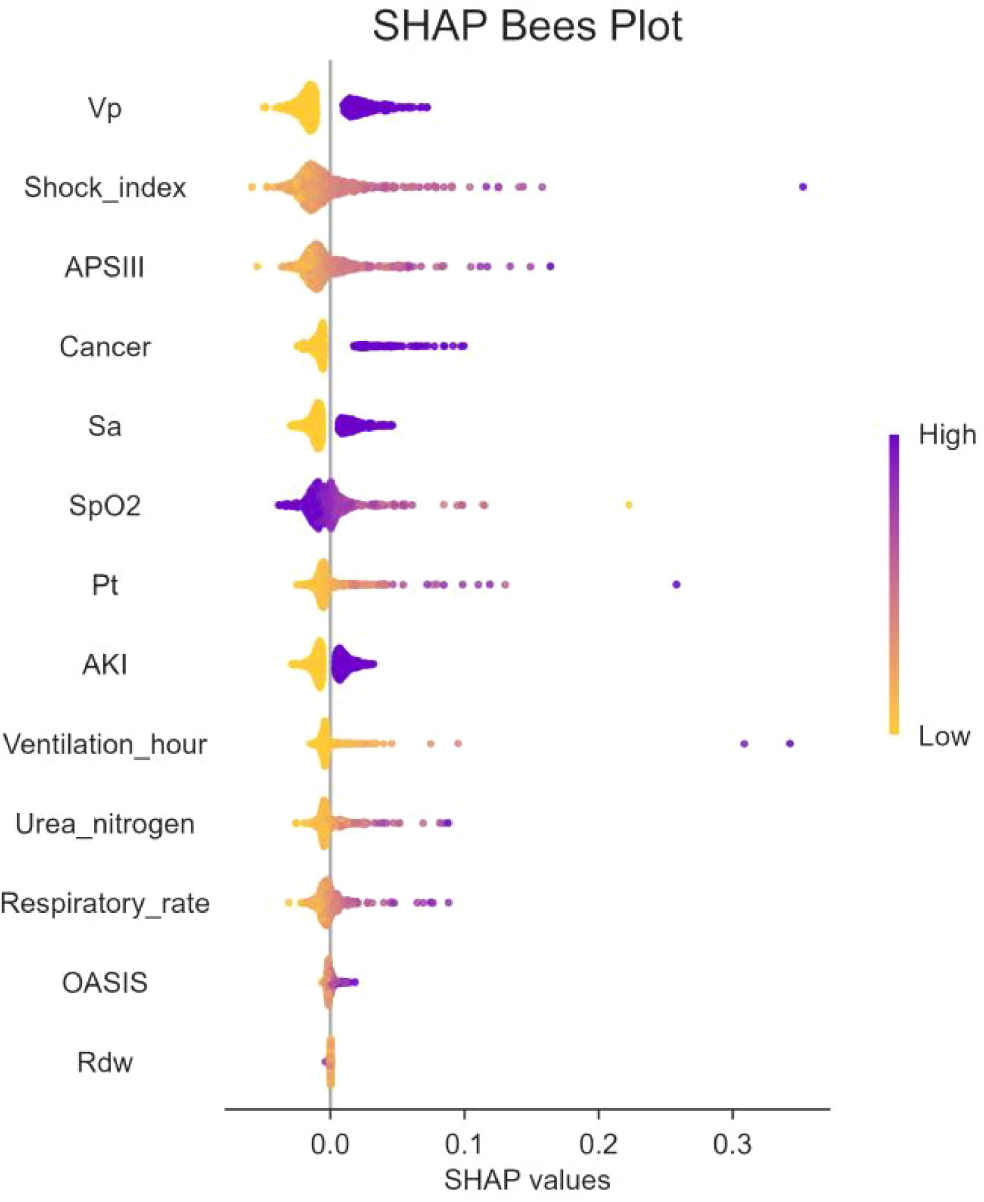
The SHapley Additive exPlanation (SHAP) values. SpO2, O2 saturation; APSIII, acute physiology and chronic health evaluation III; VP, vasopressor; SA, sedative snalgesic; PT, prothrombin time; AKI, acute kidney injury; OASIS, oxford acute illness severity score; Rdw, red blood cell distribution width; SHAP, SHapley Additive exPlanation.

In addition, SHAP values were used to identify predictor variables that had a significant effect on mortality risk and to determine their positive or negative association with the target outcome. As shown in Figure 5, the horizontal position indicates whether the effect of the value is associated with an increase or decrease in the predicted value, while the color indicates the high or low state of the variable in a particular observation.

### SHAP heat force plots

**FIGURE 6:**
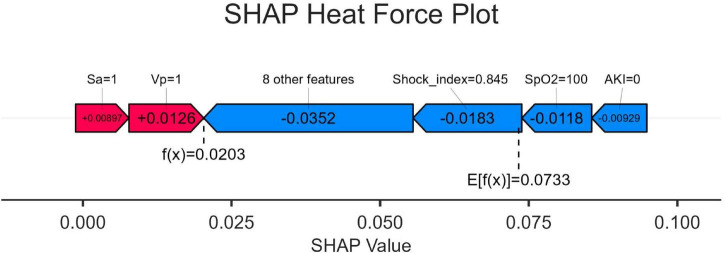
SHapley Additive exPlanation (SHAP) force plot. SpO2, O2 saturation; VP, vasopressor; SA, sedative analgesic; AKI, acute kidney injury; SHAP, SHapley Additive exPlanation.

Figure 6 shows the heat force plots for patients who did not survive and survived. The SHAP values provide insights into the predictive factors of individual patients and quantify the impact of each factor on mortality prediction. The numbers highlighted in bold represent the probabilistic predictions (f(x)), while the base values indicate the predictions made by the model without any input. The log odds ratio of each observation is represented by the function f(x). The left side displays red features that are associated with an elevated risk of mortality, while the blue features represent factors linked to a reduced risk of mortality. The magnitude of the effect on the prediction can be easily visualized by observing the length of the arrows.

## Discussion

In this study, we used a comprehensive intensive care unit (ICU) database to perform a retrospective cohort analysis. We focused on the development and validation of four different machine-learning algorithms that effectively predicted mortality in patients diagnosed with catheter-associated urinary tract infection (CAUTI). XGBoost, DT, and RF were all outperformed by the logistic regression (LR) model. The area under the curve (AUC) of the Logistic regression model was 0.871, which showed better prediction performance among the four models. The DecisionTree machine had limited generalization ability, with an AUC of 0.542 and relatively poor prediction accuracy. The poor performance of decision tree models may be related to overfitting, and its complex branch structure has limited generalization ability in small samples. Random Forest and XGBoost were prone to overfitting and calibration drift because the effective number of events was insufficient to stabilize their large parameter spaces. The superior performance of logistic regression may be due to the linear separability of CAUTI mortality prediction and its resistance to overfitting in small samples. In order to ensure the interpretability of the logistic regression model while maintaining its performance, we adopted the SHAP method for interpretation. This will enhance the understanding of the decision-making process of the model by healthcare professionals and facilitate the practical application of the predicted results. It was observed that within this range, logistic regression showed superior performance. In the field of intensive care research, logistic regression has gained significant popularity due to its application in predicting patient mortality during hospitalization, thus potentially helping healthcare professionals to make informed decisions (19–21).

It is essential to evaluate the advantages of early mortality prediction in clinical practice. In this study, 545 adult patients were included from 1344 CAUTI patients diagnosed in the MIMIC-IV database. The mortality of CAUTI patients in intensive care unit (ICU) was 7.89% (43/545). We utilized SHAP to elucidate the LR model and identify key factors associated with in-hospital mortality in CAUTI patients. Shock index, use of vasoactive drugs, concomitant malignancy, and APSIII score were identified as variables with high predictive significance. SHAP risk threshold can help early identification of high-risk patients, and it is recommended to integrate it into the early warning system of ICU electronic medical record.

However, relatively few studies have investigated the risk factors for mortality in patients with catheter-associated urinary tract infection (CAUTI). A high shock index indicates possible hemodynamic instability and is associated with increased mortality in critically ill patients (3). This instability reflects the inability of the body to maintain adequate perfusion and oxygenation of organs, which impairs their function and leads to multiple organ failure, especially in the context of infections such as CAUTI (22). The use of vasoactive drugs usually indicates the presence of severe inflammation and significant cardiovascular damage in patients, and may lead to an increase in CAUTI mortality (5). Patients with malignancies often have compromised immune systems due to the disease itself or treatment options such as chemotherapy and radiotherapy, making them more susceptible to infections, including CAUTI. Studies have shown (23) that patients with cancer face a high incidence of CAUTI, which is associated with an increased risk of death associated with these infections, and that the metabolic activity of the tumor and the potential to develop neutropenia further complicate the treatment of such patients and increase the risk of serious complications. Malignancy is an independent risk factor for 28-day mortality in patients with CAUTI. APSIII is a scoring system that assesses disease severity based on various physiological parameters; higher scores are associated with an increased risk of death in critically ill patients and can be used as a predictor of clinical outcomes (24). In the future, bedside CDSS tools can be developed to generate death risk scores by entering physiological parameters in real time. However, due to the lack of an external validation cohort, further studies are needed to explore the applicability of this research approach.

## Limitations

The strength of our research is attributed to the use of a large sample size obtained from the MIMIC database, and the statistical results are quite persuasive. However, there are several limitations in this study. Firstly, since our data were taken from a publicly accessible database, some variables were incomplete. Secondly, all data originated from ICU patients in the MIMIC database, which raises questions about how well our model can be applied to other populations. Thirdly, our mortality prediction models relied on information available within the first 24 h of each ICU admission; this may overlook subsequent events that could alter prognosis and introduce confounding factors to some degree. Lastly, due to the absence of an external validation cohort, the effectiveness of the developed LR model in clinical practice may be limited.

## Conclusion

This study provides a methodological basis for the development of a real-time prediction tool for mortality risk in the ICU and demonstrates the utility of artificial intelligence in accurately predicting catheter-associated urinary tract infection (CAUTI) and mortality in patients admitted to the intensive care unit (ICU). We created an interpretable logistic regression prediction model that performed best in assessing the risk of death in patients with CAUTI. Moreover, this interpretable machine learning approach enables effective identification of risk factors associated with CAUTI patients and will help healthcare providers to identify CAUTI patients with high mortality risk, enabling them to take timely and effective treatment measures.

## Data Availability

The original contributions presented in this study are included in this article/supplementary material, further inquiries can be directed to the corresponding author.
